# Retinal phenotyping of variants of Alzheimer's disease using ultra‐widefield retinal images

**DOI:** 10.1002/dad2.12232

**Published:** 2021-08-20

**Authors:** Lajos Csincsik, Nicola Quinn, Keir X. X. Yong, Sebastian J. Crutch, Tunde Peto, Imre Lengyel

**Affiliations:** ^1^ Wellcome‐Wolfson Institute for Experimental Medicine Queen's University Belfast Belfast UK; ^2^ Centre for Public Health Queen's University Belfast Belfast UK; ^3^ Dementia Research Centre UCL Queen Square Institute of Neurology University College London London UK

**Keywords:** Alzheimer's disease, drusen, peripheral reticular pigmentary degeneration, posterior cortical atrophy, reticular pseudodrusen, retinal imaging, retinal phenotype, sub‐retinal deposit, ultra‐widefield imaging

## Abstract

**Background:**

Posterior cortical atrophy (PCA) is the most common atypical variant of Alzheimer's disease (AD). Changes associated with PCA in the brain affect the visual cortex, but little is known about retinal changes in PCA. In this study, we explored retinal phenotypic variations in typical AD (tAD) and PCA.

**Methods:**

Retinal phenotyping was carried out on ultra‐widefield (UWF) images of 69 control, 24 tAD, and 25 PCA participants.

**Results:**

Individuals with tAD (odds ratio [OR] = 2.76 [confidence interval (CI):1.24 to 6.10], *P *= .012) and PCA (OR = 3.40 [CI:1.25 to 9.22], *P *= .016) were more likely phenotyped as hard drusen. tAD (OR = 0.34 [CI:0.12 to 0.92], *P *= .035) were less likely to have soft drusen compared to control. Almost 3‐fold increase in reticular pseudodrusen formation in tAD (OR = 2.93 [CI:1.10 to 7.76], *P *= .030) compared to control was estimated.

**Discussion:**

Studying the peripheral retina may contribute to a better understanding of differences in retinal phenotypes of different AD variants.

## Graphical abstract

Workflow for the Retinal phenotyping of variants of Alzheimer's disease using ultra widefield retinal images study.

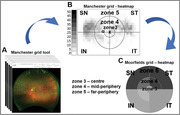

## 1 INTRODUCTION

Neuropathological and structural heterogeneity enables detailed characterization of clinical variants of Alzzheimer's disease (AD), such as typical AD (tAD), logopenic variant, behavioral/dysexecutive variant,^1^ corticobasal syndrome, and posterior cortical atrophy (PCA).^2^, ^3^

PCA is a neurodegenerative syndrome, and it is considered the most common atypical variant of AD.^2^ In tAD, the primarily affected cortical area is the medial temporal lobe. In PCA, which is often called the visual variant of AD, the primarily affected areas are the parietal, occipital, and occipitotemporal cortices.^4^, ^5^ Little is known about retinal changes in PCA despite the significant visual anomalies accompanying the disease. Recently, we investigated retinal changes in a cohort with tAD and PCA using optical coherence tomography (OCT).^6^ We found no significant differences in retinal thickness in the posterior pole (macula and optic disc) in patients compared to cognitively normal controls, despite the apparent differences observed on brain imaging.^6^

Pathological changes, however, can occur outside the posterior pole, involving the peripheral retina. Using ultra‐widefield (UWF) imaging, we reported an increased accumulation of extracellular deposits called drusen under the retinal pigment epithelium (sub‐RPE) in the peripheral retina in AD,^7^ and laboratory observations also reported peripheral retinal changes in the neurosensory retina.^8^

Deposit formation between the RPE and the retina (sub‐retinal) called reticular pseudodrusen (RPD) has been associated with outer retinal atrophy.^9^ Peripheral reticular pigmentary degeneration (PRPD), another retinal imaging feature, is associated with compromised systemic circulation and choroidal vascular insufficiency.^10^ These, and sub‐RPE deposits, are features that can be readily identified on UWF images.^11^, ^12^ We propose that monitoring these retinal changes on UWF images could help improve patient stratification in AD. In this study, we examined patients with tAD and PCA using UWF images and compared these to controls to identify retinal phenotypic differences.

## 2 METHODS

We enrolled 29 patients with PCA, 26 patients with tAD, and 72 cognitively healthy control. Participants were recruited from a tertiary specialist center, the University College London (UCL) Dementia Research Centre (DRC), between 2014 and 2016.

Participant groups were well matched for demographic characteristics, and there was no evidence of between‐group differences in age or sex (Table 1). Control did not show evidence of cognitive impairment as assessed by Mini‐Mental State Examination (MMSE ≥29; Table 1).

**TABLE 1:** Study characteristics

Characteristics	Control *N* = 69	tAD *N* = 24	PCA *N* = 25	*P*
Age: y (mean (SD))	66.53 (7.53)	63.74 (7.33)	66.37 (7.21)	.272*
MMSE (mean (SD))	29.49 (0.79)	20.00 (5.13)	22.20 (4.87)	<.001*
Sex; Males (N(%))	30 (43.5)	15 (62.5)	11 (44.0)	.255^†^
Ungradable area^‡^ (mean (SD))	32.49 (16.4)	27.16 (8.78)	33.89 (14.5)	.068*

Abbreviations: MMSE, Mini‐Mental State Examination; PCA, posterior cortical atrophy; SD, standard deviation; tAD, typical Alzheimer's disease.

* Analysis of variance (ANOVA) for continuous variables.

^†^χ^2^ test for categorical variable.

^‡^Ungradable are: one unit = one square of the MaG (Manchester grid).

PCA and tAD patients were assessed by consultant neurologists with expertise in cognitive neurology and fulfilled consensus criteria for PCA and National Institute on Aging–Alzheimer's Association (NIA‐AA) criteria for tAD.^2^, ^13^ PCA patients fulfilled Mendez et al.^14^ and Tang‐Wai et al.^15^ proposed clinical criteria based on available information at baseline visit and expert retrospective clinical review. In a subset of participants (PCA = 18; tAD = 14), the clinical assessment was also confirmed by biomarker evidence using positron emission tomography (PET) and cerebrospinal fluid (CSF), and it fulfilled the amyloid PET and CSF AD profile criteria.^16^

Ethical approval was provided by the National Research Ethics Service Committee London Queen Square; all participants provided written informed consent.

**HIGHLIGHTS**

- First ultra‐widefield retinal imaging study that shows improved patient stratification
- Both tAD and PCA associated to increased peripheral hard drusen formation
- tAD associated to decreased peripheral soft drusen formation
- tAD associated to increased peripheral reticular pseudodrusen formation

**RESEARCH IN CONTEXT**

1. **Systematic review**: Based on reviewing the available literature, we identified only one study assessing retinal changes in Alzheimer's disease (AD) using ultra‐widefield imaging. We could also identify only one study assessing retinal changes in posterior cortical atrophy using optical coherence tomography. These are our earlier publications and are appropriately cited in our article.
2. **Interpretation**: Studying the peripheral retina may contribute to a better patient stratification through more precise retinal phenotypic characterization in different variants of AD. This could support future dementia trials by reducing disease heterogeneity.
3. **Future directions**: Future clinical studies should include retinal imaging biomarkers for patient stratification and disease progression and should look beyond the macula to better understand the link between retinal and brain pathologies.

**FIGURE 1:**
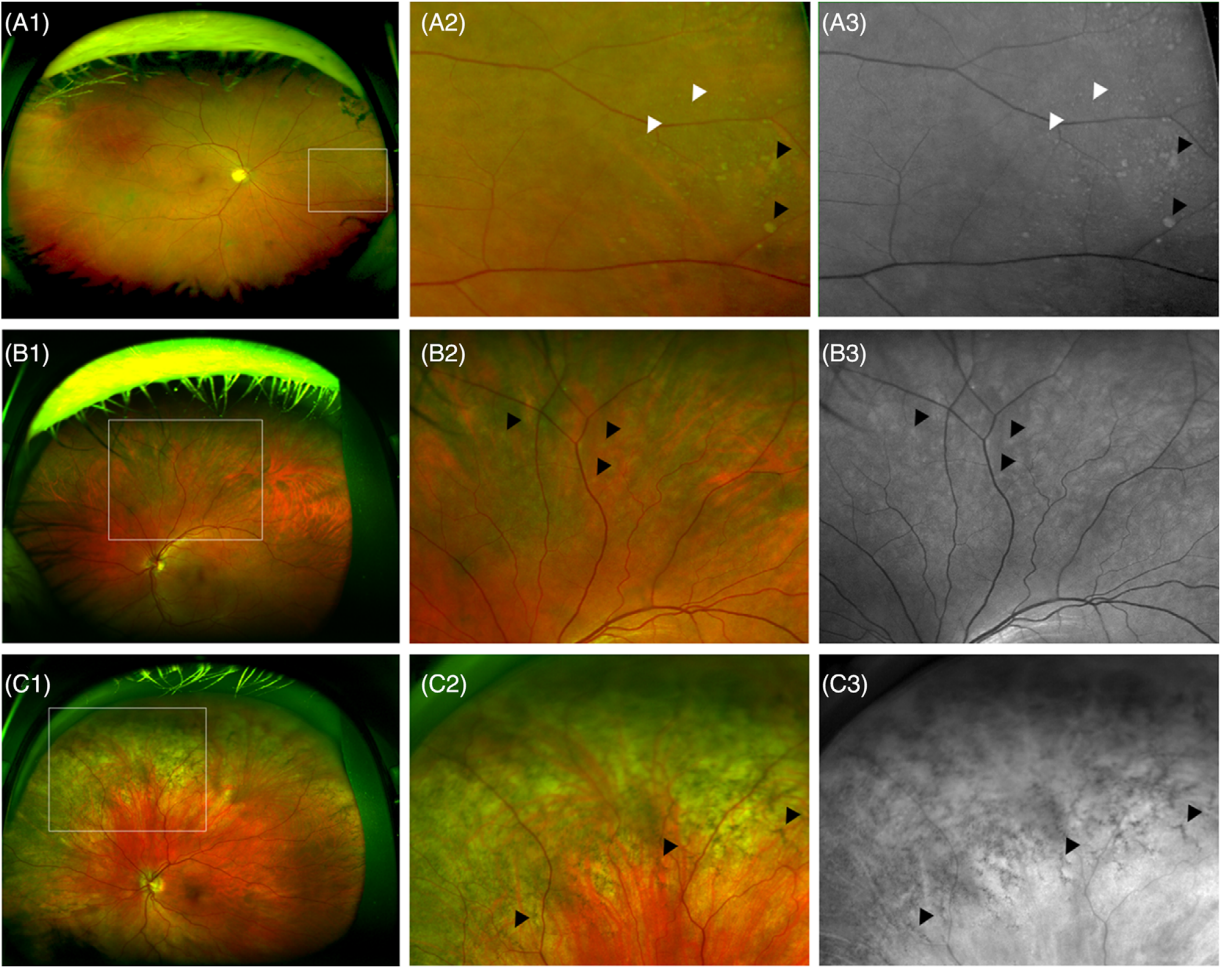
Representative ultra‐widefield images used for grading. Ultra‐widefield composite (A1 and A2) and red‐free (A3) image of a mixture of hard (white arrowheads) and soft (black arrowheads) drusen. The area outlined on A1 on the nasal periphery is visible on A2 and A3. Ultra‐widefield composite (B1 and B2) and red‐free (B3) image showing RPD (reticular pseudodrusen) at the superior hemisphere (black arrowheads). The area outlined on B1 is visible on B2 and B3. Ultra‐widefield composite (C1 and C2) and green‐free (C3) image showing PRPD (peripheral reticular pigmentary degeneration; black arrowheads). The area outlined on C1 in the superonasal periphery can be seen on C2 and C3. Drusen and RPD were often assessed using the red‐free images, while PRPD was often evaluated using the green‐free images as these give better contrast between the pathology and the rest of the image

Color UWF images were acquired using the Optomap P200Tx SLO (Optos Plc) without pupil dilation. After quality control (QC), images deemed acceptable for grading were stereographically projected to compensate for distortions due to the retinal curvature using the Optos Projection Tool.^17^ After projection, images were graded for hard and soft drusen, RPD, PRPD, geographic atrophy (GA), pigment epithelial detachment (PED), and choroidal neovascularization (CNV), retinal phenotypes that are readily detectable on UWF images.^12^ Sub‐RPE deposits, appearing as yellowish patches on color images and gray patches on red‐free images, were graded as hard (< 125 μm in size) or as soft drusen (> 125 μm)^18^ (Figure 1A1‐3). RPD (also known as sub‐retinal drusenoid deposit [SDD])^19^ was defined as mottled yellow interlacing reticular pattern on color images, and light grey similar pattern on red‐free images that ranged in size from 125  to 250 μm (Figure 1B1‐3). PRPD were coarse, netlike brown geometric patterns on color images, and dark gray corresponding patterns on red‐free images (Figure 1C1‐3). PRPD is also referred to as peripheral reticular degeneration (PRD).^12^

Images with questionable pathological changes were adjudicated by a retinal specialist (T.P.). Two independent graders carried out the grading (N.Q. and L.C.) masked to the participants’ case‐control status.

The high spatial resolution grading (Figure S1 in supporting information) was carried out after overlaying a grid of squares (that will be referred to as Manchester Grid [MaG]) in which the area of all squares (754 squares per image) equaled the size of the optic disc, using the Manchester grid tool (Optos, version r6076).^20^ During grading, each square was assessed for the presence (1) or absence (0) of the different pathological features outlined above. A square was defined as ungradable if more than 50% of the square area was impossible to assess.

**FIGURE 2:**
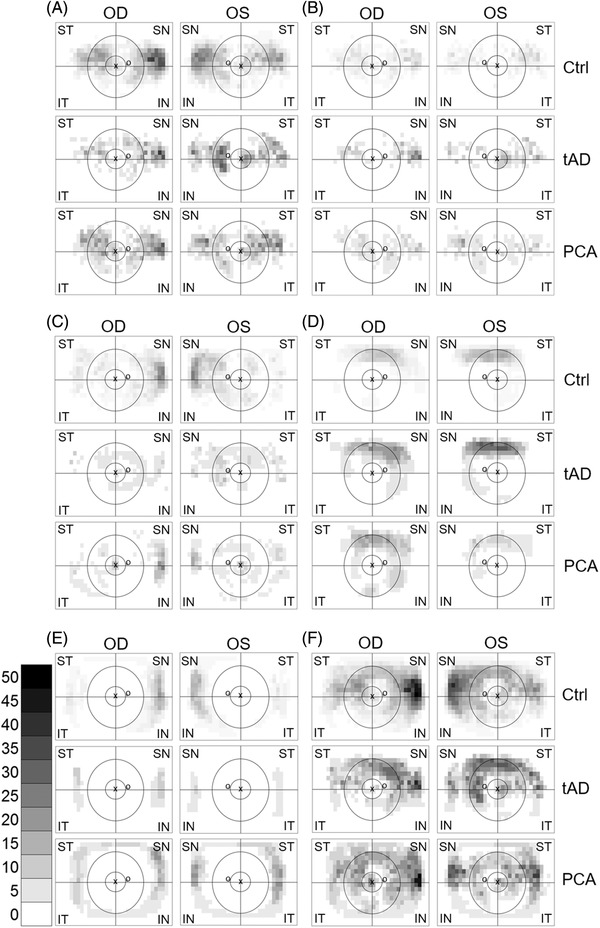
Manchester heatmaps—distribution of retinal pathologies. The heatmaps show the percentage of participants with the given square of the MaG (Manchester grid) positive for hard drusen before (A) and after (B) hierarchical phenotyping, followed by soft drusen (C), RPD (reticular pseudodrusen [D]), PRPD (peripheral reticular pigmentary degeneration [E]), and all the above pathologies on one heatmap (F). Grayscale bar shows the percentage and the corresponding shades of gray. The foveola location labelled with “x” and the optic disc labelled with “o.” Black rings and lines represent the MoG (Moorfields grid) superimposed on the Manchester heatmap for easier interpretation. Central circle represents zone 3, followed by the middle ring for zone 4 and the area beyond for zone 5. The zones are divided into four quadrants (superotemporal, inferotemporal, inferonasal, and superonasal). Ctrl, Control; tAD, typical Alzheimer's disease; PCA, posterior cortical atrophy; OD, oculus dextra (right eye); OS, oculus sinistra (left eye); ST, superotemporal; IT, inferotemporal; IN, inferonasal; SN, superonasal

**FIGURE 3:**
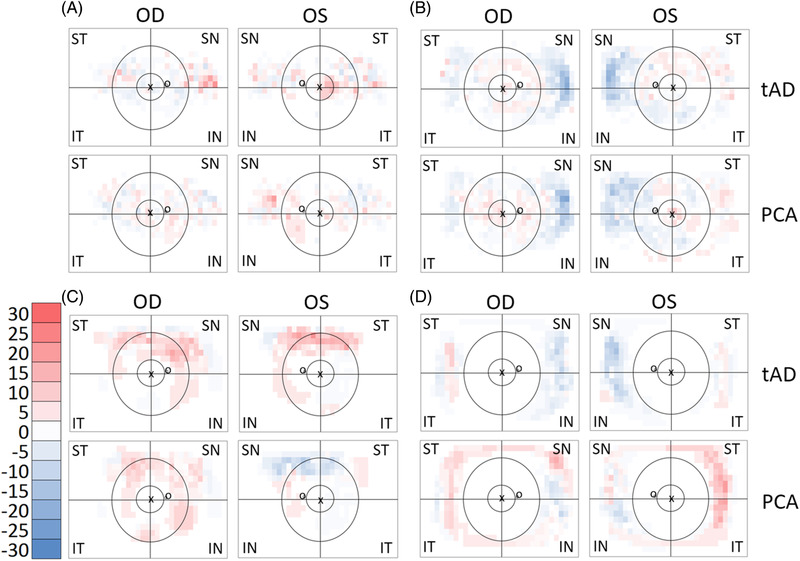
Manchester heatmap—group differences. The heatmaps show the differences in the prevalence of hard drusen after hierarchical phenotyping (A), soft drusen (B), RPD (reticular pseudodrusen [C]) and PRPD (peripheral reticular pigmentary degeneration [D]) between study groups for each square of the MaG (Manchester grid). The scale bar shows the percentage and the corresponding colors (red higher and blue lower prevalence of pathology in tAD or PCA compared to Ctrl; white, no difference). The location of the foveola labelled with “x” and the optic disc with “o.” Black rings and lines represent the MoG (Moorfields grid) superimposed on the Manchester heatmap for easier interpretation. Central circle represents zone 3, followed by the middle ring for zone 4 and the area beyond for zone 5. The zones are divided into four quadrants (superotemporal, inferotemporal, inferonasal and superonasal). Ctrl, Control; tAD, typical Alzheimer's disease; PCA, posterior cortical atrophy; OD, oculus dextra (right eye); OS, oculus sinistra (left eye); ST, superotemporal; IT, inferotemporal; IN, inferonasal; SN, superonasa

After data extraction and summation, heatmaps were generated for visual assessment for the regional distribution of pathologies in the back of the eye (Figure 2). The results from patients with tAD or PCA were subtracted from the prevalence in control. These differential plots are shown in Figure 3. Blue indicates features higher in control, while red indicates features higher in patients.

To compare the phenotypes obtained using the MaG with our earlier publication,^7^ the output from MaG grading was converted into the so‐called Moorfields grid (MoG)^12^ using a script we generated in R (v.1.1.456, 2009‐2018 RStudio, Inc.). If a square on the MaG that was mapped onto the MoG zones/quadrants was graded for a pathology, then that whole zone/quadrant was graded to have that pathology.

**FIGURE 4:**
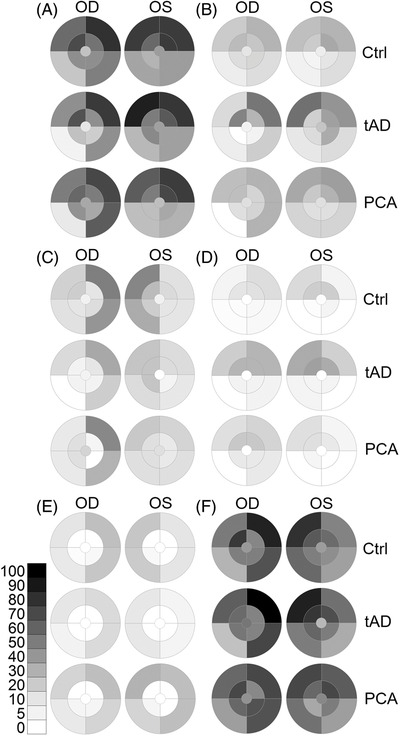
Moorfields heatmaps—distribution of retinal pathologies. Moorfields heatmaps show the zone‐ and quadrant‐specific prevalence of hard drusen before (A) and after (B) hierarchical phenotyping, followed by soft drusen (C), RPD (reticular pseudodrusen [D]), PRPD (peripheral reticular pigmentary degeneration [E]) and all the above pathologies on one heatmap (F). The central circle represents zone 3, followed by the middle ring for zone 4 and the outer ring for zone 5. Zones are divided into four quadrants (superotemporal, inferotemporal, inferonasal, and superonasal). The grayscale bar shows the percentage and the corresponding shades of gray (white, no pathology present). Ctrl, control; tAD, typical Alzheimer's disease; PCA, posterior cortical atrophy; OD, oculus dextra (right eye); OS, oculus sinistra (left eye)

**FIGURE 5:**
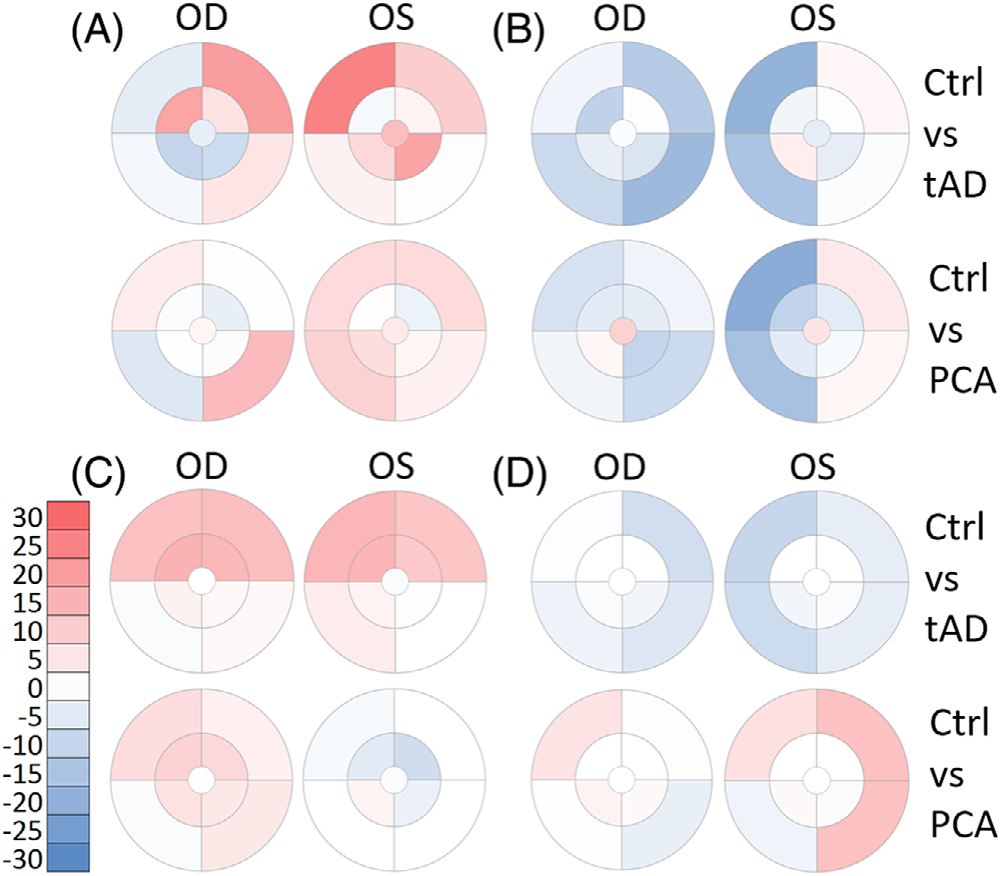
Moorfields heatmaps—group differences. The heatmaps show the differences in the prevalence of hard drusen after hierarchical phenotyping (A), soft drusen (B); RPD (reticular pseudodrusen [C]), PRPD (peripheral reticular pigmentary degeneration [D]) between study groups for each zone and quadrant of the MoG (Moorfields grid). The central circle represents zone 3, followed by the middle ring for zone 4 and the outer ring for zone 5. Zones are divided into four quadrants (superotemporal, inferotemporal, inferonasal, and superonasal). The scale bar shows the percentage and the corresponding colors (red higher and blue lower prevalence of pathology in tAD or PCA compared to Ctrl; white, no difference). Ctrl, control; tAD, typical Alzheimer's disease; PCA, posterior cortical atrophy; OD, oculus dextra (right eye); OS, oculus sinistra (left eye)

The zones and sectors of the MoG were designed based on the original definition of a standard macular grid defined by the distance between the centers of the optic nerve head and the fovea and extended by zone 4 for the mid periphery and zone 5 for the far periphery.^12^ Zones 1, 2, and 3, corresponding to the Early Treatment Diabetic Retinopathy Study (ETDRS) grid^21^ of the central retina, were merged into one central zone, labeled as zone 3. The workflow is depicted in Figure S1. After conversion, heatmaps for all grading categories were plotted for distribution (Figure 4) and differences (Figure 5).

For direct comparison with our previous study,^7^ we carried out hierarchical phenotyping as well: Those with hard drusen only were assigned to the hard drusen phenotype; those with both hard and soft drusen were designated as soft drusen phenotype.

### 2.1 Data analysis

All data analysis was conducted using SPPS (version 26.0; SPSS Inc.). When assessing differences in study characteristics, the Chi‐square test was used for categorical variables and one‐way analysis of variance for continuous variables. General estimating equation (GEE) enabled data from both eyes to be included in the binary logistic regression analysis,^22^ which was used to assess the relationship between retinal pathologies and diagnosis, with control as a reference group. The size of the ungradable area was recorded for each image as a continuous variable and included as a covariate in the final GEE analysis. For all comparison, an alpha level of *P* < .05 was used.

Intergrader agreement was calculated using kappa (k) statistics and interpreted according to Landis and Koch.^23^

A total of six participants (three control, one tAD, and two PCA) could not be imaged due to machine failure. Images of three participants (one tAD and two PCA) could not be graded due to artefacts caused by participant misalignment, making the final participant number 118 (69 control, 24 tAD, and 25 PCA). In the case of five tAD and two PCA participants, only one eye could be graded due to artefacts or media opacities in one of the eyes. Overall, 229 eyes (138 control, 43 tAD, and 48 PCA) were phenotyped in this study.

## 3 RESULTS

### 3.1 Prevalence of pathologies

The difference in ungradable area size on UWF images between the three groups did not reach statistical significance (*P* = .068; Table 1, Figure S2 in supporting information).

Four eyes in the control, two eyes in the tAD, and one eye in the PCA group showed no detectable pathological changes on our UWF images (Table 2). Hard drusen were detected in 133 eyes of 69 control, 41 eyes of 23 tAD, and 46 eyes of 25 PCA participants (Table 2). Seventy‐nine eyes of 47 control, 17 eyes of 13 tAD, and 24 eyes of 16 PCA had soft drusen (Table 2). After hierarchical phenotyping, 55 eyes of 35 control, 24 eyes of 15 tAD, and 23 eyes of 15 PCA remained in the hard drusen phenotype, and the others were graded as soft drusen phenotype (Table 2). RPD was detected in 23 eyes of 15 control, 16 eyes of 10 tAD, and 8 eyes of 6 PCA (Table 2). PRPD was present in 41 eyes of 25 control, 6 eyes of 4 tAD, and 16 eyes of 10 PCA participants (Table 2). There was no GA, PED, or CNV detected in any of the eyes in this study (Table 2).

**TABLE 2:** Pathological findings

	Control		tAD		PCA	
	*N* = 138	*N* = 69	*N* = 43	*N* = 24	*N* = 48	*N* = 25
Pathology	Eye (%)	Participant (%)	Eye (%)	Participant (%)	Eye (%)	Participant (%)
No pathology	4 (2.9)	0 (0.0)	2 (4.7)	1 (4.2)	1 (2.1)	0 (0.0)
Hard druse**^a^**	133 (96.4)	69 (100.0)	41 (95.3)	23 (95.8)	46 (95.8)	25 (100.0)
Hard druse**^b^**	55 (39.9)	35 (50.7)	24 (55.8)	15 (62.5)	23 (47.9)	15 (60.0)
Soft druse	79 (57.2)	47 (68.1)	17 (39.5)	13 (54.2)	24 (50.0)	16 (64.0)
RPD	23 (16.7)	15 (21.7)	16 (37.2)	10 (41.7)	8 (16.7)	6 (24.0)
PRPD	41 (29.7)	25 (36.2)	6 (14.0)	4 (16.7)	16 (33.3)	10 (40.0)
GA	0 (0.0)	0 (0.0)	0 (0.0)	0 (0.0)	0 (0.0)	0 (0.0)
PED	0 (0.0)	0 (0.0)	0 (0.0)	0 (0.0)	0 (0.0)	0 (0.0)
CNV	0 (0.0)	0 (0.0)	0 (0.0)	0 (0.0)	0 (0.0)	0 (0.0)

Notes: The table shows the number (and corresponding percentage) of eyes and participants unaffected or affected by the given pathology.

Abbreviations: CNV, choroidal neovascularization; GA, geographic atrophy; pCA, posterior cortical atrophy; PED, pigment epithelial detachment; PRPD, peripheral reticular pigmentary degeneration; RPD, reticular pseudodrusen; tAD, typical Alzheimer's disease.

^a^Prevalence of hard drusen before hierarchical phenotyping. ^b^Prevalence of hard drusen after hierarchical phenotyping.

### 3.2 Distribution of pathological features using the MaG

Sub‐RPE deposits were distributed with a preference toward zone 5, especially at the superonasal (SN) and temporal quadrants (Figure 2A‐C). While hard drusen were the most frequent pathological feature, after hierarchical phenotyping, the distribution of the hard‐drusen‐only phenotype was detected in the nasal periphery, primarily in zone 5 (Figure 2B). Similarly, soft drusen were most prevalent in the nasal far‐periphery in zone 5 (Figure 2C).

RPDs were distributed mainly on the superior retinal quadrants in zones 4 and 5 (Figure 2D). PRPD featured mainly at the nasal retinal far‐periphery (zone 5; Figure 2E), although in PCA, there was a distinct ring appearance of PRPD in zone 5. When all pathologies were combined, we found that retinal pathologies were present throughout the retina with an enrichment in the far nasal periphery (Figure 2F).

Kappa statistics showed moderate agreement between graders for all the graded pathologies, with PRPD showing the highest (κ = .573) and hard drusen the lowest (κ = .440) agreement between the two graders.

To identify disease‐specific changes, tAD and PCA patients’ prevalence values were subtracted from those of control (Figure 3). The prevalence of hard‐drusen‐only phenotype was higher in patients than control (Figure 3A). However, the prevalence of soft drusen was lower in patients than in control, especially in the nasal quadrants (Figure 3B). RPD prevalence was higher in tAD in the superior quadrants, while a mixed picture can be seen in PCA (Figure 3C). PRPD was higher in PCA, especially in the temporal quadrants, while it appeared lower in tAD, especially in the nasal quadrants (Figure 3D).

### 3.3 Distribution of pathological features using the MoG

Similar to our previous findings, the prevalence of pathologies was higher in the peripheral retina, especially in the nasal quadrants (Figure 4). To better visualize the differences between patients and control, we subtracted the prevalence values from those in control and plotted the differences in Figure 5. Hard drusen had the highest prevalence in Zone 5 in the SN quadrant in tAD (Figure 5A). The prevalence of peripheral soft drusen was lower in the nasal quadrants, both in tAD and PCA (Figure 5B). In tAD, PDR was higher in the superior quadrant with little difference observed in PCA (Figure 5C). In contrast, the prevalence of PRPD was higher in zone 5 in PCA but lower in tAD (Figure 5D).

Using GEE analysis, we found that individuals with tAD were twice as likely to have hard drusen phenotype in zone 5 compared to the control group (odds ratio [OR] = 2.24 [confidence interval (CI): 1.00 to 4.98], *P *= .048; Table 3, Figure S3 in supporting information), a difference likely to be driven by the difference in the SN quadrant (OR = 2.76 [CI: 1.24 to 6.10], *P *= .012; Table 3, Figure S3). PCA patients were more likely to have hard drusen phenotype in the inferonasal (IN) quadrant of zone 5 (OR = 3.40 [CI: 1.25 to 9.22], *P *= .016; Table 3, Figure S3).

**TABLE 3:** GEE analysis for sub‐RPE deposit

	tAD^a^		PCA^a^		tAD^b^		PCA^b^	
Pathology	OR (95% CI)	*P* value	OR (95% CI)	*P* value	OR (95% CI)	*P* value	OR (95% CI)	*P* value
HD‐G	1.72 (0.78 to 3.82)	.177	1.39 (0.65 to 2.97)	.396	1.79 (0.79 to 4.01)	.157	1.37 (0.64 to 2.95)	.411
HD‐ Z3	1.66 (0.59 to 4.64)	.333	1.48 (0.46 to 4.74)	.503	1.82 (0.62 to 5.29)	.271	1.47 (0.45 to 4.79)	.515
HD‐ Z4	1.56 (0.67 to 3.61)	.296	1.00 (0.45 to 2.20)	.996	1.64 (0.70 to 3.85)	.249	0.98 (0.44 to 2.16)	.966
HD ‐ Z4 ST	1.62 (0.71 to 3.69)	.251	0.89 (0.45 to 1.76)	.737	1.77 (0.76 to 4.12)	.183	0.86 (0.43 to 1.70)	.668
HD ‐ Z4 IT	1.41 (0.54 to 3.66)	.478	1.07 (0.30 to 3.74)	.915	0.62 (0.22 to 1.72)	.362	0.96 (0.27 to 3.44)	.961
HD ‐ Z4 IN	0.87 (0.22 to 3.44)	.845	1.48 (0.47 to 4.70)	.498	0.83 (0.20 to 3.38)	.800	1.50 (0.47 to 4.76)	.491
HD ‐ Z4 SN	1.06 (0.40 to 2.84)	.896	0.90 (0.34 to 2.38)	.831	1.05 (0.39 to 2.80)	.910	0.90 (0.33 to 2.41)	.840
HD ‐ Z5	2.33 (1.05 to 5.16)	**.037**	1.43 (0.68 to 3.03)	.344	2.24 (1.00 to 4.98)	**.048**	1.45 (0.68 to 3.10)	.330
HD ‐ Z5 ST	1.13 (0.47 to 2.70)	.777	1.40 (0.63 to 3.10)	.402	1.13 (0.47 to 2.70)	.780	1.40 (0.63 to 3.11)	.402
HD ‐ Z5 IT	0.88 (0.22 to 3.50)	.863	0.79 (0.25 to 2.51)	.700	0.98 (0.24 to 3.92)	.984	0.79 (0.24 to 3.92)	.697
HD ‐ Z5 IN	1.51 (0.44 to 5.23)	.508	2.82 (1.11 to 7.20)	**.029**	1.31 (0.35 to 4.81)	.679	3.40 (1.25 to 9.22)	**.016**
HD ‐ Z5 SN	2.97 (1.35 to 6.52)	**.007**	1.25 (0.59 to 2.62)	.554	2.76 (1.24 to 6.10)	**.012**	1.30 (0.60 to 2.78)	.500
SD‐G	0.53 (0.23 to 1.22)	.138	0.74 (0.34 to 1.63)	.466	0.49 (0.21 to 1.13)	.098	0.76 (0.34 to 1.67)	.499
SD‐ Z3	0.47 (0.05 to 4.05)	.494	2.74 (0.78 to 9.59)	.115	0.45 (0.52 to 3.98)	.477	2.78 (0.78 to 9.93)	.115
SD‐ Z4	0.58 (0.22 to 1.52)	.273	0.59 (0.22 to 1.56)	.597	0.57 (0.22 to 1.51)	.266	0.59 (0.22 to 1.56)	.295
SD ‐ Z4 ST	0.53 (0.14 to 2.02)	.357	0.57 (0.17 to 1.85)	.353	0.55 (0.14 to 2.13)	.394	0.56 (0.17 to 1.82)	.340
SD ‐ Z4 IT	.040 (0.05 to 3.33)	.403	1.00 (0.28 to 3.50)	.992	0.41 (0.05 to 3.46)	.419	1.00 (0.28 to 3.45)	1.000
SD ‐ Z4 IN	0.95 (0.30 to 2.98)	.935	0.34 (0.09 to 1.22)	.101	0.90 (0.28 to 2.83)	.859	0.32 (0.09 to 1.13)	.859
SD ‐ Z4 SN	0.88 (0.29 to 2.67)	.834	0.40 (0.10 to 1.52)	.180	0.85 (0.28 to 2.54)	.779	0.41 (0.10 to 1.54)	.410
SD ‐ Z5	0.40 (0.16 to 0.96)	**.042**	0.50 (0.22 to 1.12)	.096	0.37 (0.15 to 0.92)	**.033**	0.52 (0.23 to 1.16)	.112
SD ‐ Z5 ST	0.92 (0.27 to 3.06)	.892	0.87 (0.29 to 2.61)	.816	0.94 (0.27 to 3.24)	.934	0.86 (0.29 to 2.57)	.796
SD ‐ Z5 IT	0.38 (0.08 to 1.75)	.219	0.94 (0.27 to 3.22)	.924	0.35 (0.74 to 1.72)	.201	0.97 (0.28 to 3.35)	.961
SD ‐ Z5 IN	0.39 (0.15 to 1.03)	.058	0.49 (0.19 to 1.28)	.149	0.30 (0.11 to 0.84)	**.022**	0.59 (0.21 to 1.61)	.303
SD ‐ Z5 SN	0.37 (0.14 to 0.96)	**.042**	0.41 (0.16 to 1.05)	.064	0.34 (0.12 to 0.92)	**.035**	0.43 (0.17 to 1.07)	.071

Notes: Binary logistic regression analysis using GEE, assessing the relationships between the presence of sub‐RPE deposit in different sectors of the MoG (Moorfields grid) and diagnosis (tAD, PCA) with control as a reference group, Abbreviations: CI, confidence interval; G, global (entire image); GEE, generalized estimating equation; HD, hard druse; IN, inferonasal quadrant; IT, inferotemporal quadrant; OR, odds ratio; PCA, posterior cortical atrophy; SD, soft druse; SN, superonasal quadrant; ST, superotemporal quadrant; tAD, typical Alzheimer's disease; Z4, zone 4; Z5, zone 5.

^a^Unadjusted and ^b^adjusted for adjusted for ungradable area size. Bold numbers indicate statistical significance (*P* < .05).

The prevalence of soft drusen appeared to be lower in both tAD and PCA (Figure 5B), a difference that reached statistical significance only in zone 5 in tAD (OD = 0.37 [CI: 0.15 to 0.92], *P *= .033) compared to control, especially in the SN (OR = 0.30 [CI: 0.11 to 0.84], *P *= .022) and IN (OR = 0.34 [CI: 0.12 to 0.92], *P *= .035) quadrants (Table 3, Figure S3).

The prevalence of RPD appeared to be significantly higher in tAD (OR = 2.93 [CI: 1.10 to 7.76], *P *= .030) compared to control, especially in zone 5 (OR = 3.06 [CI: 1.12 to 8.37], *P *= .029), a difference driven by the superotemporal (ST) quadrant (OR = 5.19 [CI: 1.35 to 9.86], *P *= .016; Table 4, Figure S3). There was no significant difference detected in RPD between PCA and control (Table 4, Figure S3).

**TABLE 4:** GEE analysis for reticular pseudodrusen and peripheral reticular pigmentary degeneration

	tAD^a^		PCA^a^		tAD^b^		PCA^b^	
Pathology	OR (95% CI)	*P* value	OR (95% CI)	*P* value	OR (95% CI)	*P* value	OR (95% CI)	*P* value
RPD‐G	2.98 (1.12 to 7.91)	**.028**	0.97 (0.33 to 2.80)	.955	2.93 (1.10 to 7.76)	**.030**	1.03 (0.35 to 3.03)	.944
RPD‐ Z4	2.69 (0.99 to 7.33)	.052	1.02 (0.35 to 2.98)	.969	2.67 (0.99 to 7.19)	.051	1.10 (0.37 to 3.28)	.855
RPD‐ Z4 ST	2.47 (0.88 to 6.93)	.086	0.97 (0.30 to 3.09)	.967	2.50 (0.91 to 6.84)	.074	1.08 (0.33 to 3.51)	.887
RPD‐ Z4 IT	1.58 (0.25 to 9.91)	.626	1.43 (0.22 to 9.05)	.698	1.46 (0.23 to 8.94)	.682	1.58 (0.25 to 9.83)	.624
RPD‐ Z4 IN	2.04 (0.17 to 24.58)	.573	2.92 (0.45 to 18.69)	.257	2.06 (0.17 to 24.73)	.566	3.48 (0.52 to 23.19)	.196
RPD‐ Z4 SN	2.57 (0.91 to 7.28)	.074	1.07 (0.36 to 3.18)	.893	2.60 (0.93 to 7.30)	.068	1.19 (0.40 to 3.53)	.752
RPD‐ Z5	3.08 (1.24 to 8.48)	**.029**	1.21 (0.41 to 3.59)	.721	3.06 (1.12 to 8.37)	**.029**	1.32 (0.44 to 3.90)	.613
RPD‐ Z5 ST	5.50 (1.36 to 22.18)	**.017**	1.94 (0.38 to 9.83)	.420	5.19 (1.35 to 9.86)	**.016**	1.98 (0.41 to 9.41)	.388
RPD‐ Z5 IT	N/A	N/A	N/A	N/A	N/A	N/A	N/A	N/A
RPD‐ Z5 IN	3.24 (0.28 to 37.09)	.343	2.93 (0.41 to 20.78)	.281	3.18 (0.27 to 36.35)	.351	3.17 (0.45 to 22.14)	.244
RPD‐ Z5 SN	2.63 (0.94 to 7.40)	.065	1.04 (0.36 to 2.99)	.938	2.64 (0.95 to 7.33)	.061	1.11 (0.38 to 3.23)	.846
PRPD‐ G	0.39 (0.12 to 1.30)	.127	1.13 (0.46 to 2.77)	.783	0.39 (0.12 to 1.30)	.127	1.14 (0.47 to 2.80)	.763
PRPD‐ Z5	0.41 (0.12 to 1.34)	.141	1.17 (0.48 to 2.87)	.722	0.40 (0.12 to 1.34)	.141	1.18 (0.48 to 2.89)	.704
PRPD‐ Z5 ST	0.76 (0.15 to 3.88)	.751	2.24 (0.70 to 7.18)	.173	0.72 (0.14 to 3.68)	.721	2.35 (0.74 to 7.44)	.146
PRPD‐ Z5 IT	0.43 (0.51 to 3.65)	.439	1.85 (0.57 to 5.97)	.302	0.41 (0.04 to 3.52)	.419	1.74 (0.54 to 5.59)	.348
PRPD‐ Z5 IN	0.45 (0.11 to 1.73)	.247	0.72 (0.23 to 2.21)	.575	0.45 (0.11 to 1.73)	.250	0.75 (0.25 to 2.31)	.628
PRPD‐ Z5 SN	0.39 (0.10 to 1.50)	.173	1.17 (0.42 to 3.24)	.755	0.39 (0.10 to 1.51)	.177	1.20 (0.43 to 3.31)	.721

Notes: Binary logistic regression analysis using GEE, assessing the relationships between the presence of RPD and PRPD in different sectors of the Moorfields grid and diagnosis (tAD, PCA) with control as a reference group.

Abbreviations: CI, confidence interval; G, global (entire image); GEE, generalized estimating equation; HD, hard druse; IN, inferonasal quadrant; IT, inferotemporal quadrant; OR, odds ratio; PCA, posterior cortical atrophy; SD, soft druse; SN, superonasal quadrant; ST, superotemporal quadrant; tAD, typical Alzheimer's disease; Z4, zone 4; Z5, zone 5.

^a^Unadjusted and ^b^adjusted for ungradable area size. Bold numbers indicate significant (*P* < .05) association.

While on the visual representations there appeared to be an increased prevalence of PRPD in PCA, but not in tAD compared to control (Figure 5D), the difference did not reach statistical significance in any of the zones or quadrants (Table 4, Figure S3).

The same results were obtained when the model was or was not adjusted for ungradable area size, apart from soft drusen in the IN quadrant of zone 5, when tAD was compared to control. In this case, a significant difference was only detected in the adjusted final GEE analysis.

## 4 DISCUSSION

We have previously shown that peripheral retinal hard drusen were associated with AD.^7^ This study expanded on this finding. We analyzed UWF images using a higher resolution grading grid to identify potential “hot spots” for pathology. This more detailed grading then was transformed into the low‐resolution grid we used previously.^7^ We included retinal phenotypes that were not considered earlier, and it appears that RPD and PRPD could become distinct phenotypes to stratify patients with AD.

Undoubtedly, the higher resolution MaG grading grid allowed us to generate more nuanced maps of pathological changes on UWF images (Figures 2 and 3). However, developing these maps was time consuming and resource intensive and may not be feasible in a clinical setting unless automated grading algorithms are developed.

In the current study, patients were stratified into typical AD and PCA, based on consensus criteria for PCA^2^ and NIA‐AA criteria for tAD.^13^ Despite dividing patients into variants, we found that both patients with tAD and PCA had a higher prevalence of hard druse‐only phenotype in the far periphery, especially in the SN quadrant, supporting our previous findings on a general AD population.^7^ We also found a lower prevalence of soft drusen phenotype at the retinal far‐periphery in tAD, but not in PCA, compared to control. A trend for lower soft drusen prevalence was also present in the general AD population.^7^ Drusen is considered a hallmark for age‐related macular degeneration (AMD), and lipids and apolipoprotein E (*apoE*) have been associated with drusen formation and progression.^24^, ^25^, ^26^ Although the exact role of *apoE* in AMD is still under intense investigation,^27^, ^28^, ^29^ the difference in drusen load might be linked to the higher *apoE* ε4 allele frequency in tAD^30^ compared to PCA.^31^

Overall, the prevalence of drusen phenotypes was higher in this study than what we reported earlier.^7^ This difference might be accounted for by the detailed grading carried out using the MaG in the current study compared to the previous study in which the MoG was used with the adopted AREDS study protocol^32^ in which at least 10 drusen had to be present in the given sector of the MoG to grade the sector positive for drusen.^7^

Whether the more detailed grading used in this study could be introduced into clinical practice will need to be elucidated in subsequent studies. The mapping of the MaG data onto the MoG suggested no appreciable clinical advantage, though this does not consider the benefit of using this detailed grid in follow‐up studies.

We believe that this is the first study to assess RPD for AD. RPD (also known as SDD) was first recognized by Arnold et al. in *post mortem* tissues with AMD.^33^ Later, it was shown that RPD was associated with AMD in vivo and conferred an increased risk to progress to late AMD.^34^ In population‐based studies and cohorts of AMD patients, RPD prevalence showed a considerable variation.^35^, ^36^, ^37^, ^38^ In our study, the prevalence of RPD in control and PCA were ≈16% but significantly higher in patients with tAD (37.2%). This finding appears to associate RPD‐like pathology with tAD, especially in the superior hemisphere in the peripheral retina. The molecular mechanism of RPD formation is not yet fully elucidated.^39^ It is clear that RPDs are different from sub‐RPE deposits,^39^ but how the molecular composition of RPD might be linked with tAD and what the difference in retinal phenotype between tAD and PCA means is yet to be fully explored. RPD can be detected using OCT, which shows an accumulation of debris internal to the retinal pigment epithelium in the macula.^39^ The detection of peripheral and far peripheral changes with current OCT cameras, however, is challenging.

A small cross‐sectional study identified retinal features that are positive for curcumin and most commonly present in the superior periphery of the retina in patients with AD.^8^ After assessing these lesions using OCT, the authors could not rule out the possibility that the curcumin signal originates from RPD.^8^

The recent development of a UWF‐guided swept‐source OCT, which enables OCT imaging at any part of the retina, giving a more comprehensive analysis of the retinal periphery,^40^ might help define the role of the observed high prevalence of RPD in the peripheral retina in tAD.

Another distinguishing feature between tAD and PCA was the prevalence of PRPD. While there was no evidence for statistically significant differences in PRPD prevalence between groups in our study, given the ease with which this feature can be detected on UWF images,^12^ this retinal phenotype may be an attractive candidate for a specific biomarker in larger cohorts. The clinical importance of PRPD is somewhat incomplete.^10^ A cross‐sectional, observational study using UWF fluorescein angiography, established a link between compromised systemic and choroidal circulation and the development of PRPD.^10^ Choroidal^41^, ^42^, ^43^ and retinal^7^, ^44^, ^45^, ^46^ vascular changes have been associated with AD, which may explain the observed retinal phenotypic variation between groups in our study. However, choroidal and retinal vascular changes yet to be explored in PCA.

There are some limitations of our study. The cross‐sectional design and the relatively small number of participants may potentially limit the statistical power. While we carried out a detailed analysis of the images, we do not have detailed medical information on all patients. Diabetes, hypertension, or smoking^47^, ^48^ are just a few factors that may contribute to changes in retinal phenotypes we reported here. Also, the intergrader agreement was moderate for all categories in this study. While this is not unusual in image grading studies,^49^, ^50^ the outcome highlights human grading's potentially subjective nature, especially for sub‐RPE deposit.

Our study's strengths are that our cohort only included well‐characterized tAD and PCA patients, and these were compared to a sizeable control population. In addition, we graded all eyes with sufficient quality and used GEE analysis that appropriately accounts for the correlation between the two eyes,^22^ providing a more comprehensive analysis. Also, we used a high‐resolution grading grid to refine our phenotypes.

In summary, our study highlighted the need for better patient stratification through more precise phenotypic characterization, which could include UWF imaging of the retina. These could help the success of future dementia trials to reduce disease heterogeneity. We should look beyond the macula to better understand the link between retinal and brain pathologies.

Support received none relevant to the submitted work: ATHENA; NIHR; NIHR HTA; NIHR EME; NIHR CEAT; Alcon UK, via South Tyneside and Sunderland NHS Foundation Trust; FFS; Novartis; Reading centre component – Queen's University Belfast; Alcon Eye Care UK Ltd; MRC research grant (MR/N029941/1); Horizon 2020 grant; Fight for Sight; Macular Society; Special Trustees of Moorfields Eye Hospital; Queen's Diamond Jubilee Trust Fund; Lowy Medical Research Institute; DFE‐GCRF Global Research Fund; Bayer; Novartis; Heidelberg; Alimera; Oxurion; Roche.

## CONFLICTS OF INTEREST

LC was supported by an unrestricted PhD studentship from Optos plc. LC is currently employed by Optos plc.

## Supporting information

Supporting material
