# Supplementary material for: Retinal phenotyping of variants of Alzheimer's disease using ultra‐widefield retinal images
Source: Alzheimers Dement (Amst). 2021 Aug 20;13(1):e12232. doi: 10.1002/dad2.12232 (PMC8377778; doi:10.1002/dad2.12232)
Supplement: Supplementary file 1 — Supporting material [file DAD2-13-e12232-s001.docx]

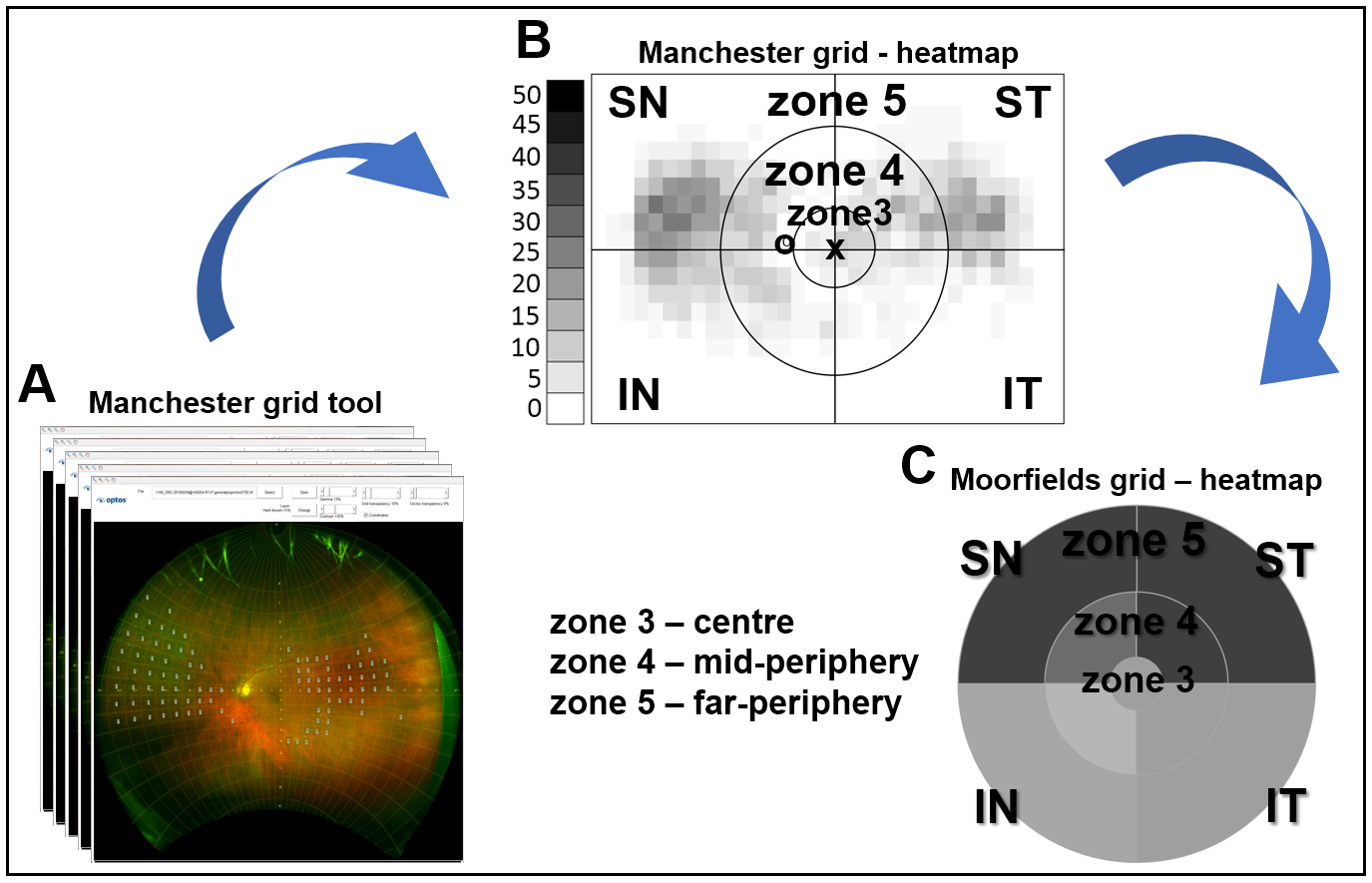


Figure S1 Methodology explained

Manchester grid tool with the MaG (Manchester grid) superimposed on a ultra-widefield image (A). Squares with the white dots represent squares with the given pathology present. Representative image of Manchester heat map showing the percentage of participants positive for the given pathology in the given square of the grid across the entire retina (B). Black rings and lines represent the MoG (Moorfields grid) overlaid onto the Manchester heatmap (O=optic disk, x=fovea) for easier interpretation. After Manchester to Moorfields data conversion, bar charts were generated, and statistical analysis was undertaken according to the sectors of the MoG. Representative image of Moorfields heatmap showing the prevalence of pathology for each quadrant (ST, superotemporal; IT, inferotemporal; IN, inferonasal; SN, superonasal) across the three zones (zone 3, 4 and 5) (C).


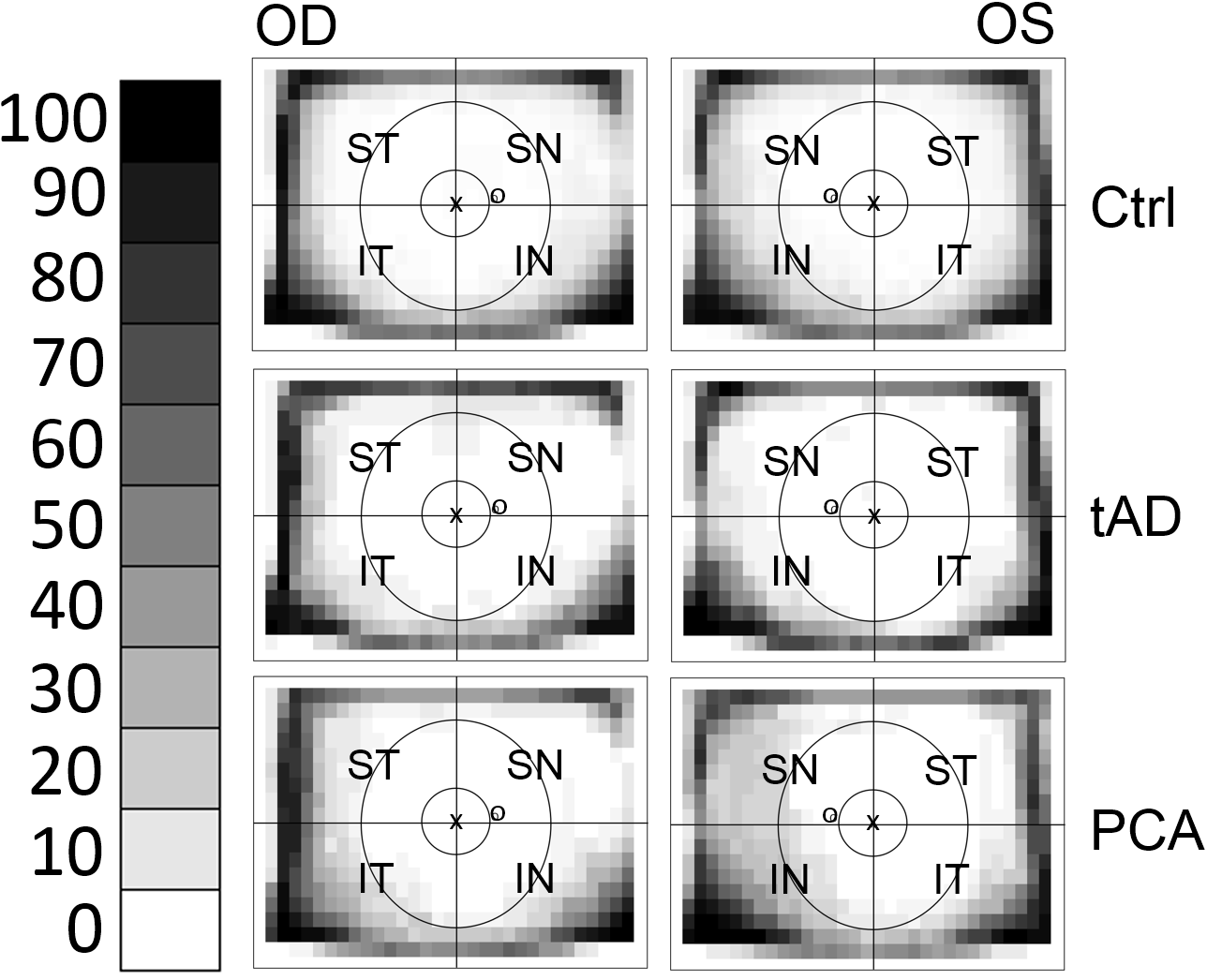


Figure S2 Manchester heatmap – ungradable area

The heatmaps show the percentage of participants with the given square of the MaG (Manchester grid) ungradable for each participant group. The greyscale bar shows the percentage and the corresponding shades of grey. The foveola location is labelled with "x" and the optic disc is labelled with "o". Black rings and lines represent the MoG (Moorfields grid) superimposed on the Manchester heatmap for easier interpretation. Abbreviation: Ctrl, Control; tAD, typical Alzheimer's disease; PCA, Posterior cortical atrophy; OD, oculus dextra (right eye); OS, oculus sinistra (left eye); ST, superotemporal; IT, inferotemporal; IN, inferonasal; SN, superonasal;


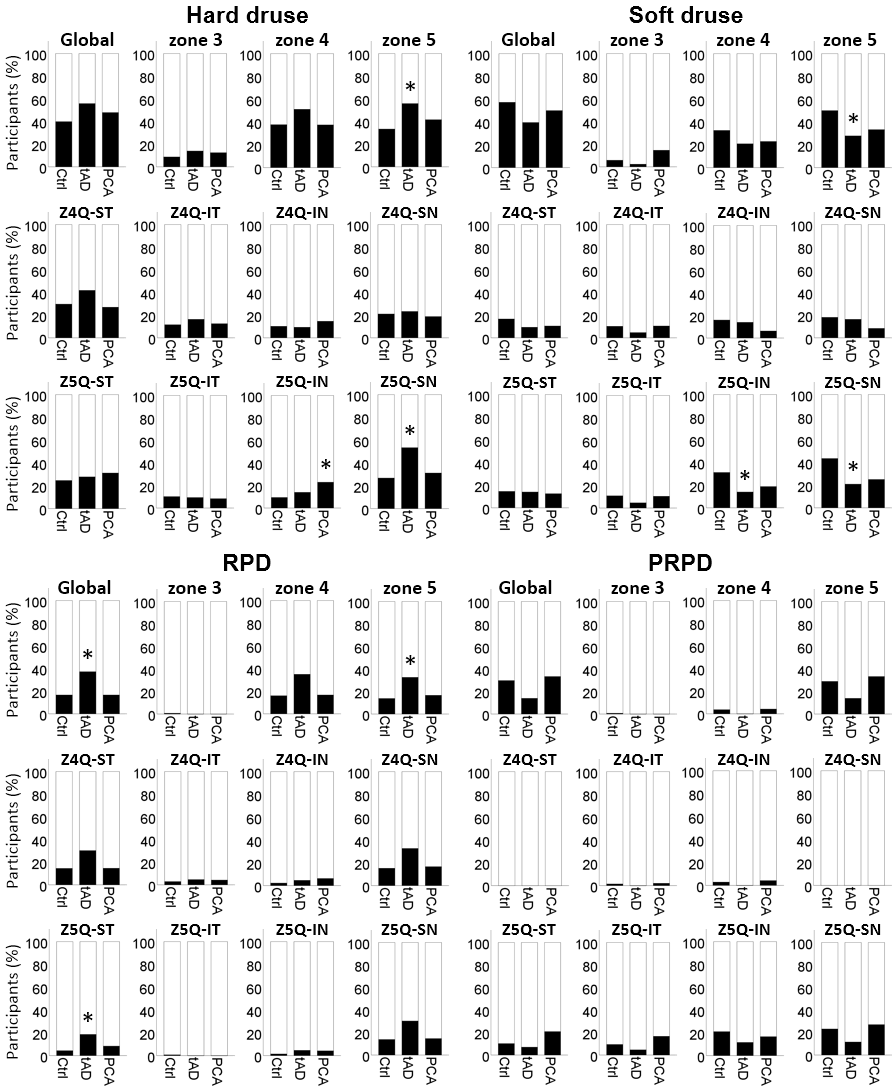


Figure S3 Prevalence of AMD-like pathologies

Bar charts show the percentage of participant positive for the given pathology across zones and quadrants of the MoG (Moorfields grid) for each participant group. Asterisk labels a significant association between the exposer and the outcome with control as a reference arm. Abbreviation: Ctrl, control; tAD, typical Alzheimer's disease; PCA, Posterior cortical atrophy; Z, zone; Q, quadrant; RPD, reticular pseudodrusen; PRPD, peripheral reticular pigmentary degeneration; ST, superotemporal; IT, inferotemporal; IN, inferonasal; SN, superonasal;
